# The Impact of Tumor Mutation Burden on the Effect of Frontline Trastuzumab Plus Chemotherapy in Human Epidermal Growth Factor Receptor 2-Positive Advanced Gastric Cancers

**DOI:** 10.3389/fonc.2021.792340

**Published:** 2021-12-02

**Authors:** Hye Ryeon Kim, Soomin Ahn, Hyunji Jo, Hongsik Kim, Joohyun Hong, Jeeyun Lee, Ho-Yeong Lim, Won Ki Kang, Seung Tae Kim

**Affiliations:** ^1^ Division of Hematology-Oncology, Department of Medicine, Samsung Medical Center, Sungkyunkwan University School of Medicine, Seoul, South Korea; ^2^ Department of Internal Medicine, Dong-A University College of Medicine, Busan, South Korea; ^3^ Department of Pathology, Samsung Medical Center, Sungkyunkwan University School of Medicine, Seoul, South Korea

**Keywords:** trastuzumab, advanced gastric cancer, HER2, tumor mutational burden, biomarker

## Abstract

**Background:**

Trastuzumab is a HER2-trargeted humanized monoclonal antibody that has been studied as a first-line treatment for patients with HER2-positive advanced gastric cancer (AGC). The effect of anti-HER2 therapy according to tumor mutational burden (TMB) in HER2-positive AGC remains unclear.

**Methods:**

We performed next-generation sequencing (NGS), including TMB analysis, in 31 HER2-positive AGC patients with trastuzumab plus chemotherapy as first-line therapy for recurrent (n=8) or metastatic (n=23) tumors. The TruSight Oncology 500 Assay from Illumina (San Diego, CA, USA) was used to evaluate TMB.

**Results:**

Among 31 patients, 30 had tumors with immunohistochemistry (IHC) 3+, and one was IHC 2+ and silver *in situ* hybridization (SISH) positive. The median age was 57.0 years old (range, 35-76), and the majority had tumors with low TMB (87.1%, n=27/31). Only four (12.9%) had tumors with high TMB. Of these four, three achieved complete response (CR) or partial response (PR) to treatment, and the remaining patient was not evaluable for tumor response. Objective response rate (ORR) to trastuzumab plus chemotherapy showed a favorable trend in patients with high TMB (75.0%, n=3/4) compared to patients with low TMB (59.3%, n=16/27) (*P*=0.546). The median progression-free survival (PFS) was not reached in the TMB-high group but was 8.0 months (95% CI, 7.6-8.5) in the TMB-low group (*P*=0.019)

**Conclusion:**

The status of TMB could be a novel biomarker in predicting the efficacy of trastuzumab plus chemotherapy in HER2-positive AGCs.

## Introduction

Gastric cancer (GC) is the sixth most common cancer and has the third-highest global mortality rate in 2018 (1). In Korea, the stomach is the second most common site of cancer following lung, and GC is the fifth-highest mortality following lung, liver, colorectal, and pancreas cancers (2). The frequency of human epidermal growth factor receptor 2 (HER2) overexpression in gastric and gastroesophageal cancer varies by study, with a mean of 18%, and is a significant predictor of poor survival (3–5). Trastuzumab, a monoclonal antibody targeting HER2, inhibits HER2-mediated signaling and prevents cleavage of the extracellular domain of HER2 (6). In a previous study, trastuzumab plus chemotherapy showed a survival advantage in HER2-positive advanced gastric and gastroesophageal junction cancers (7). However, the overall response rate (ORR) was 47%, with a complete response (CR) of only 5%, and the available data demonstrated that high dose trastuzumab is not associated with efficacy improvement (7, 8). Thus, there is a need for potential biomarkers regarding the efficacy of anti-HER2 therapy.

Tumor mutational burden (TMB) means the total number of mutations per coding area of a tumor genome. There is a strong relationship between the TMB and the activity of immune checkpoint inhibitors (ICIs) across multiple cancers (9–12). Hu et al. suggested that TMB could predict the response to trastuzumab rather than HER2 status in patients with advanced gastric cancer (AGC) (13). However, few studies have been performed on the relationship between TMB and the efficacy of anti-HER2 therapies.

Herein, we analyzed the efficacy of anti-HER2 therapy in patients with HER2-positive AGC according to TMB.

## Patients and Methods

### Patients

Patients, who had started trastuzumab plus chemotherapy for metastatic or recurrent HER2-positive AGC at Samsung Medical Center between April 2019 and June 2021, were retrospectively analyzed. The patients had to be tested prospectively for molecular aberrations, including TMB, with the TruSight Oncology 500 assay. Either metastatic or recurrent tumor tissues had to show HER2 positivity as IHC 3+ or IHC 2+ with ERBB2 gene amplification by silver *in situ* hybridization (SISH). The prior palliative systemic chemotherapy was not allowed, but adjuvant chemotherapy was allowed. The patients received trastuzumab, capecitabine, and cisplatin (HXP) as first-line therapy. This study was reviewed and approved by our institutional review board (IRB number: 2021-08-123-001).

### TruSight Oncology 500 Assay

Forty (40) ng of DNA was quantified with the Qubit dsDNA HS Assay (Thermo Fisher Scientific, Inc., Waltham, MA, USA) on the Qubit 2.0 Fluorometer (Thermo Fisher Scientific) and then sheared using a Coraris E220 focused-ultrasonicator (Woburn, MA, USA) and the 8 microTUBE–50 Strip AFA Fiber V2 following the manufacturer’s instructions. The treatment time was optimized for formalin fixed paraffin embedded (FFPE) material. The treatment settings were as follows: peak incident power (W): 75; duty factor: 15%; cycles per burst: 500; treatment time (s): 360; temperature (°C): 7; water level: 6. For DNA library preparation and enrichment, the TruSight Oncology 500 Kit (Illumina) was used following the manufacturer’s instructions. Post-enriched libraries were quantified, pooled, and sequenced on a NextSeq 500 (Illumina Inc., San Diego, CA, USA). The quality of the NextSeq 500 (Illumina) sequencing runs was assessed with the Illumina Sequencing Analysis Viewer (Illumina). Sequencing data were analyzed with the TruSight Oncology 500 Local App Version 1.3.0.39 (Illumina), a comprehensive tumor profiling assay designed to identify known and emerging tumor biomarkers, including small variants, splice variants, and fusions. Importantly, the TruSight Oncology 500 measures tumor mutational burden (TMB) and microsatellite instability (MSI), features that are potential key biomarkers for immunotherapy. TMB was reported as mutations per megabase (Mb) sequenced, and high TMB was defined as more than 10 mutations per Mb (≥10Mut/Mb).

### Statistics

Descriptive statistics are reported as proportion and median. Data are presented as numbers (%) for categorical variables. Response categories were assessed according to Response Evaluation Criteria In Solid Tumors (RECIST) 1.1. Duration of response (DOR) was analyzed in patients who achieved CR or PR and was calculated from the date of CR or PR to the date of progression or death. Progression-free survival (PFS) was defined as the time from the date of anti-HER2 treatment to the date of disease progression. Overall survival (OS) was defined as the time from the date of anti-HER2 treatment and the date of death from any cause. Analyses of PFS and OS were censored at the date of the last follow-up visit. The survival analysis was performed using the Kaplan-Meier method and comparative analysis was performed by the log-rank test and Fisher’s exact test. A *P* value of less than 0.05 was considered statistically significant. Statistical analysis was performed using IBM SPSS statistics version 27 (Armonk, NY, USA).

## Results

### Patients

A total of 31 patients were analyzed retrospectively in this study. The median age was 57.0 years old (range, 35-76), and the number of males and females was 25 (80.6%) and six (19.4%), respectively. Twenty-four (77.4%) patients were diagnosed with tubular adenocarcinoma, and the degree of differentiation was moderate in 16 (51.6%). All patients had microsatellite stable disease (MSS). Thirty (96.8%) patients had IHC 3+ tumors, and only one patient had IHC 2+ and SISH positive tumors. Twenty-seven (87.1%) and four (12.9%) patients had low and high TMB disease, respectively. Only one patient in the TMB-low group was positive for Epstein-Barr virus (EBV)-status. Fifteen (48.4%) had programmed death-ligand 1 (PD-L1) positivity, one in the TMB-high group and 14 in the TMB-low group. Twenty-three (74.2%) patients were first diagnosed with metastatic AGC, and eight (25.8%) patients had the recurrent disease at the time of HXP administration. Among eight with the recurred disease, one patient had locally recurred TMB-high tumor, and seven with systemic recurrence (**Table 1**).

**Table 1:** Patient characteristics.

Variables	Total (n = 31)	TMB-high (n = 4)	TMB-low (n = 27)
**Median age (y) (range)**	57.0 (35-76)	60.5 (42-76)	57.0 (35-76)
**Sex**			
Male	25 (80.6%)	4 (100%)	21 (77.8%)
Female	6 (19.4%)	–	6 (22.2%)
**Disease classification**			
Metastatic	23 (74.2%)	2 (50.0%)	21 (77.8%)
Recurrent	8 (25.8%)	2 (50.0%)^†^	6 (22.2%)
**Histologic subtype**			
Adenocarcinoma	5 (16.1%)	2 (50.0%)	3 (11.1%)
Tubular adenocarcinoma	24 (77.4%)	2 (50.0%)	22 (81.5%)
Signet ring cell carcinoma	2 (6.5%)	–	2 (7.4%)
**Differentiation**			
Well	2 (6.5%)	–	2 (7.4%)
Moderately	16 (51.6%)	2 (50.0%)	14 (51.9%)
Poorly	12 (38.7%)	2 (50.0%)	10 (37.0%)
Unknown	1 (3.2%)*	–	1 (3.7%)
**HER2**			
IHC 3+	30 (96.8%)	4 (100%)	26 (96.3%)
IHC 2+, SISH positive	1 (3.2%)	–	1 (3.7%)
**EBV**			
Positive	1 (3.2%)	–	1 (3.7%)
Negative	19 (61.3%)	2 (50.0%)	17 (63.0%)
Unknown	11 (35.5%)	2 (50.0%)	9 (33.3%)
**PD-L1**			
<1%	4 (12.9%)	–	4 (14.8%)
1-20%	15 (48.4%)	1 (25.0%)	14 (51.9%)
Unknown	12 (38.7%)	3 (75.0%)	9 (33.3%)
**MSI**			
MSI-high	–	–	–
MSS	31 (100%)	4 (100%)	27 (100%)
**Previous treatment**			
Gastrectomy	10 (32.3%)	2 (50.0%)	8 (29.6%)^‡^
Adjuvant chemotherapy	4 (12.9%)	–	4 (14.8%)^ 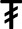 ^
Palliative radiotherapy	2 (6.5%)	4 (100%)	2 (7.4%)

*The patient was diagnosed with gastric cancer by biopsy of a metastatic brain lesion from the stomach. ^†^In the TMB-high group, two patients had recurrent disease, including one with local recurrence. ^‡^Among eight patients who had received gastrectomy, six patients received radical gastrectomy at the first diagnosis, one received radical gastrectomy after second lines of chemotherapy, and one received palliative surgery. ^₮^Adjuvant chemotherapy was completed more than 6 months before the treatment started.

TMB, tumor mutational burden; HER2, human epidermal growth factor receptor 2; IHC, immunohistochemistry; SISH, silver in-situ hybridization; EBV, Epstein-Barr virus; PD-L1, programmed death-ligand 1; MSI, microsatellite instability; MSS, microsatellite stable.

### Efficacy and Survival

The median follow-up duration was 10.8 months (range, 1.7-20.9). The median number of treatment cycles was 7.0 (range, 2-20), and the median treatment duration was 4.7 months (range, 0.7-14.2). In all patients, the ORR was 61.3%, including 1 (3.2%) CR and 18 (58.1%) of PR. Four (12.9%) patients had stable disease (SD), and the other four (12.9%) had progressive disease (PD). In four patients with TMB-high tumor, three achieved CR or PR, and the other patient was not evaluated for tumor response due to short treatment duration. In 27 patients with TMB-low tumor, ORR was 59.3%, and four (14.8%) patients had PD. The ORR to trastuzumab plus chemotherapy showed a favorable trend in patients with TMB-high tumor (75%, n=3/4) compared to patients with TMB-low tumor (59.3%, n=16/27) (*P*=0.546) (**Table 2**).

**Table 2:** Best response – No. of patients (%).

	Total patients (n = 31)	TMB-high (n = 4)	TMB-low (n = 27)
CR	1 (3.2%)	1 (25.0%)	–
PR	18 (58.1%)	2 (50.0%)	16 (59.3%)
ORR	19 (61.3%)	3 (75.0%)	16 (59.3%)
SD	4 (12.9%)	–	4 (14.8%)
PD	4 (12.9%)	–	4 (14.8%)
NE*	4 (12.9%)	1 (25.0%)	3 (11.1%)

*Four patients were prior to the first response assessment due to short treatment duration. TMB, tumor mutational burden; CR, complete response; PR, partial response; ORR, objective response rate; SD, stable disease; PD, progressive disease; NE, non-evaluable.

**Figure 1:**
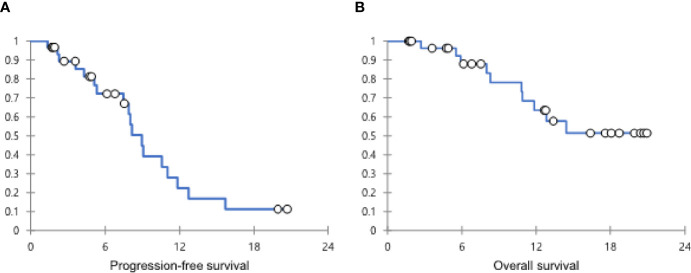
The Kaplan-Meier curves showing PFS **(A)** and OS **(B)** in all patients. PFS, progression-free survival; OS, overall survival.

**Figure 2:**
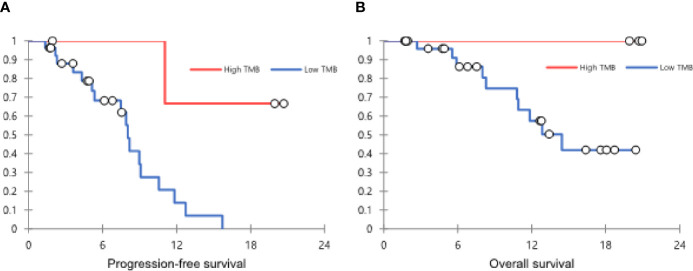
The Kaplan-Meier curves showing PFS **(A)** and OS **(B)** according to the TMB status. PFS, progression-free survival; OS, overall survival; TMB, tumor mutational burden.

**Figure 3:**
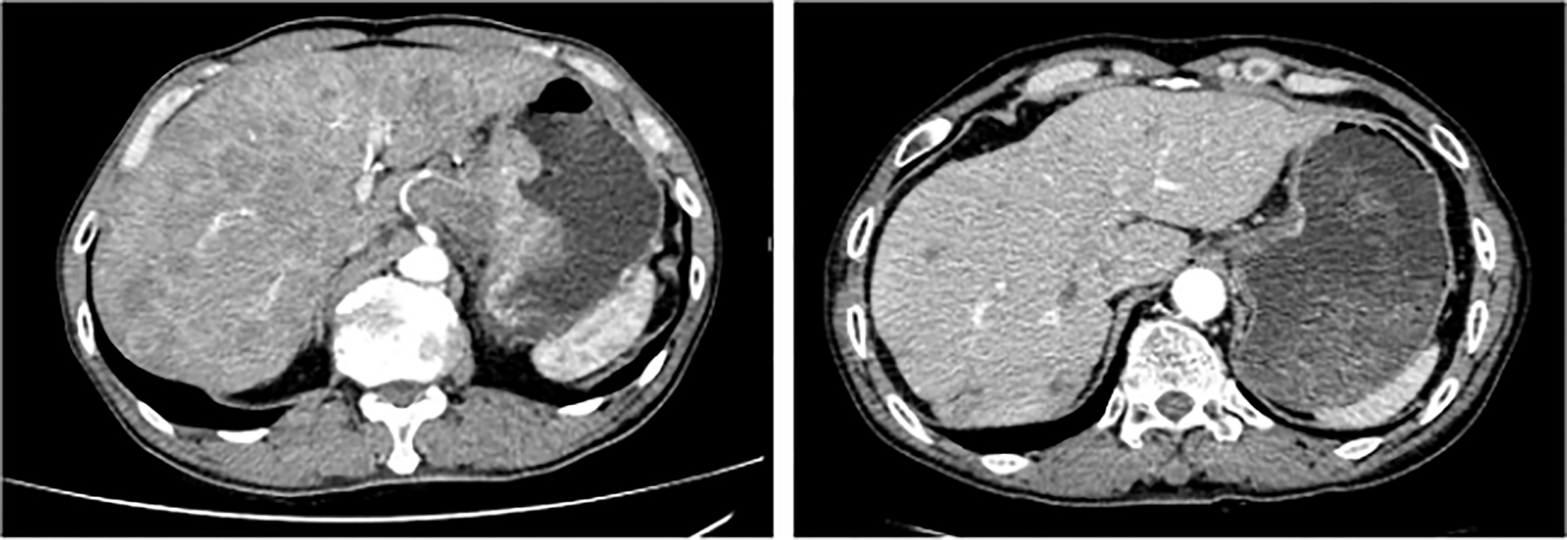
Computed tomography (CT) scan in a HER2-positive patient with high TMB achieving CR to HXP. The image on the left was at the time of treatment start, and the image on the right was at the time of CR achievement. HER2, human epidermal growth factor receptor 2; TMB, tumor mutational burden; CR, complete response; HXP, trastuzumab, capecitabine, and cisplatin.

In patients with CR or PR, the median DOR was 6.1 months (IQR, 3.4-9.6). The median PFS was 9.0 months (95% confidence interval [CI], 7.1-10.8), and the median OS was not achieved in all populations (**Figures 1A, B**). According to TMB status, the median PFS was not reached in patients with TMB-high tumor but was 8.0 months (95% CI, 7.6-8.5) in patients with TMB-low tumor (*P*=0.019) (hazard ratio [HR], 0.122; 95% CI, 0.016-0.954). The median OS was not achieved in patients with TMB-high tumor but was 14.5 months (95% CI, 10.3-18.7) in patients with TMB-low tumor (*P*=0.117) (HR, 0.034; 95% CI, 0-31.208) (**Figures 2A, B**). The computed tomography (CT) imaging before and after HXP treatment of the patient who achieved CR was presented in **Figure 3**.

## Discussion

In this study, we retrospectively analyzed the relationship between TMB and response to trastuzumab plus chemotherapy as first-line treatment in metastatic or recurrent GC. There was no difference in ORR and OS between the TMB-high and low groups (*P*=0.546 and 0.117, respectively). However, PFS in the TMB-high group was longer than that in the TMB-low group with statistically a significant difference (*P*=0.019).

GC has a molecular heterogeneity and has been classified to diffuse and intestinal subtypes according to Lauren classification (14). In 2014, The Cancer Genome Atlas (TCGA) genomically separated GC into four subtypes, such as EBV-associated tumors, microsatellite instability-high (MSI-H) tumors, genomically stable tumors, and tumors with chromosomal instability (15). In the era of immunotherapy, pembrolizumab, PD-L1 monoclonal antibody, showed a durable response rate in patients with advanced gastric or gastroesophageal cancer that had progressed after second-line treatment (16). Especially in a previous study, EBV-positive and/or MSI-high GC had a higher response to an anti-PD-L1 therapy compared to other subtypes according to TCGA (17). A recent study showed that nivolumab, the first programmed cell death-1 (PD-1) inhibitor, plus chemotherapy had superior OS and PFS with tolerable toxicity profile in patients with previously untreated advanced gastric, gastroesophageal junction, or esophageal adenocarcinoma (18). However, this study included only non-HER2-positive disease, and the importance of trastuzumab is still emphasized in patients with HER2-positive gastric or gastroesophageal cancers.

Despite the development of targeted therapy, surgical resection is still the only curable treatment option for GC, and preoperative chemo-radiotherapy or perioperative chemotherapy is needed to improve the outcomes for locally advanced disease (clinically T2-4 or positive lymph node). In the metastatic setting, the first-line chemotherapy consists of platinum-based doublet or triplet with or without trastuzumab according to HER2 status (19). In ToGA (Trastuzumab for Gastric Cancer) trial, trastuzumab plus chemotherapy showed significantly longer OS (18.6 *versus* 17.1 months; HR 0.74 [95% CI 0.60-0.91]; *P*=0.0046) than the chemotherapy alone for HER2-positive gastric or gastroesophageal junction cancer. And the ORR and CR were 47% (n=139/294) and 5% (n=16/294), respectively (7).

Considering that high TMB correlates with a greater probability of displaying tumor neoantigens on human leukocyte antigen molecules on the surface of tumor cells (20, 21), it was suggested that the tumors with higher TMB are more likely to respond to immunotherapy. In several previous studies, the benefit of high TMB on response to immunotherapy has been reported in many cancer types, including melanoma, non-small cell lung cancer, and bladder cancer (9–12). Furthermore, a previous case report suggested that TMB could be a predictor of the response to trastuzumab in patients with HER2-positive AGCs (13). However, as far as we know, there was no clinical trial to evaluate the value of TMB as a predictor of efficacy of trastuzumab in HER2-positive AGCs.

This study had several limitations. First, it had a small sample size, was retrospective in nature, and utilized a heterogeneous population, all conductive to bias. Second, in patients with low TMB, anti-HER2 therapy showed a useful effect. This suggests that the status of TMB is not a sufficient biomarker for selecting patients likely to benefit from HXP. Third, only Asian patients with HER2-positive AGC were analyzed in the study, limiting the generalizability because of differences in molecular profiles and clinical features between Western and Eastern patients with HER2-positive AGC.

Therefore, study findings for high TMB as a novel biomarker should be interpreted with caution and make it difficult to draw definite conclusions. Further prospective clinical trials are required to determine whether high TMB could be a novel predictive or prognostic biomarker for anti-HER2 therapy in HER2-positive AGC. Furthermore, as next-generation sequencing (NGS) is available in the biomarker-based trials are in practice (22).

## Data Availability Statement

The original contributions presented in the study are included in the article. Further inquiries can be directed to the corresponding author.

## Ethics Statement

The studies involving human participants were reviewed and approved by the Institutional Review Board Samsung Medical Center. Written informed consent for participation was not required for this study in accordance with the national legislation and the institutional requirements.

## Author Contributions

The conception and design of the study: HRK and STK. Acquisition of data: HRK, SA, HJ, HK, JH, JL, H-YL, WK, and STK. Analysis and interpretation of data: HRK and STK. Drafting the article or revising it critically for important intellectual content: HRK and STK. Final approval of the version to be submitted: HRK and STK. All authors contributed to the article and approved the submitted version.

## Acknowledgment

This research was supported by a grant of the Korea Health Technology R&D Project through the Korea Health Industry Development Institute (KHIDI), funded by the Ministry of Health & Welfare, Republic of Korea (grant number: HR20C0025).

## Conflict of Interest

The authors declare that the research was conducted in the absence of any commercial or financial relationships that could be construed as a potential conflict of interest.

## Publisher’s Note

All claims expressed in this article are solely those of the authors and do not necessarily represent those of their affiliated organizations, or those of the publisher, the editors and the reviewers. Any product that may be evaluated in this article, or claim that may be made by its manufacturer, is not guaranteed or endorsed by the publisher.
